# Detection of Target Genes for Drug Repurposing to Treat Skeletal Muscle Atrophy in Mice Flown in Spaceflight

**DOI:** 10.3390/genes13030473

**Published:** 2022-03-08

**Authors:** Vidya Manian, Jairo Orozco-Sandoval, Victor Diaz-Martinez, Heeralal Janwa, Carlos Agrinsoni

**Affiliations:** 1 Department of Electrical & Computer Engineering, University of Puerto Rico, Mayaguez, PR 00681-9000, USA; jairo.orozco@upr.edu (J.O.-S.); victor.diaz16@upr.edu (V.D.-M.); 2 Biomedical Engineering, University of Puerto Rico, Mayaguez, PR 00681-9000, USA; 3 Department of Mathematics, University of Puerto Rico, Rio Piedras, PR 00925-2537, USA; heeralal.janwa@upr.edu (H.J.); carlos.agrinsoni@upr.edu (C.A.)

**Keywords:** machine learning, skeletal muscle atrophy, graph convolutional neural networks, graph neural network, random forest, gradient boosting method, knowledge graphs, node embeddings, random walk, diseases, drugs, link prediction

## Abstract

Skeletal muscle atrophy is a common condition in aging, diabetes, and in long duration spaceflights due to microgravity. This article investigates multi-modal gene disease and disease drug networks via link prediction algorithms to select drugs for repurposing to treat skeletal muscle atrophy. Key target genes that cause muscle atrophy in the left and right extensor digitorum longus muscle tissue, gastrocnemius, quadriceps, and the left and right soleus muscles are detected using graph theoretic network analysis, by mining the transcriptomic datasets collected from mice flown in spaceflight made available by GeneLab. We identified the top muscle atrophy gene regulators by the Pearson correlation and Bayesian Markov blanket method. The gene disease knowledge graph was constructed using the scalable precision medicine knowledge engine. We computed node embeddings, random walk measures from the networks. Graph convolutional networks, graph neural networks, random forest, and gradient boosting methods were trained using the embeddings, network features for predicting links and ranking top gene-disease associations for skeletal muscle atrophy. Drugs were selected and a disease drug knowledge graph was constructed. Link prediction methods were applied to the disease drug networks to identify top ranked drugs for therapeutic treatment of skeletal muscle atrophy. The graph convolution network performs best in link prediction based on receiver operating characteristic curves and prediction accuracies. The key genes involved in skeletal muscle atrophy are associated with metabolic and neurodegenerative diseases. The drugs selected for repurposing using the graph convolution network method were nutrients, corticosteroids, anti-inflammatory medications, and others related to insulin.

## 1. Introduction

Spaceflight experiments using mice are being conducted to determine the impact of microgravity on different muscle groups [1]. A major health problem in spaceflight is muscle wastage due to microgravity. The primary muscles in the human body are the muscles of the upper limb and lower limb. Experiments on hind limb muscle wasting after a 13-day shuttle flight have shown reduced knee weight bearing and meniscal degradation, inducing an arthritic phenotype in cartilage and menisci [2]. Changes in electrical impedance characteristics in gastrocnemius muscles are also induced by spaceflight [3]. Skeletal muscle atrophy is a secondary effect of aging (sarcopenia) and diseases such as diabetes, cancer and kidney diseases. The primary muscles in the human body are the upper limb and lower limb. Studies have shown that muscle gene expression is different in spaceflight vs. that on the ground. Models of sarcopenia and age-related muscle loss have been studied in [4]. Spaceflight induces similar muscle loss, and the analysis of their gene expression (see [5]) has revealed that a majority of 272 mRNAs that were significantly altered by spaceflight displayed similar responses to hind limb suspension.

There are several molecular processes that influence muscle atrophy. The muscle RING-finger protein-1 that plays an important role in muscle remodeling is an E3 ubiquitin ligase expressed in skeletal and cardiac muscle tissues [6]. Spaceflight induces unique muscle atrophy in animal models. The MuRF1 nullified mice did not show improvement in soleus muscle loss, showing that atrophy proceeds under unique mechanisms in spaceflight [7]. Muscle mass is a balance between protein generation and degradation. A decreased rate of synthesis causes skeletal muscle wasting. The ubiquitin proteosome is the protein synthesis pathway in muscle atrophy. It has been shown that proteosome inhibition reduces denervation-induced muscle atrophy [8]. One of the most important muscle-wasting cytokines is tumor necrosis factor-a (TNF-a), elevated levels of which cause significant muscular abnormalities. Although there has been some advancement in understanding cellular and molecular mechanisms such as MuRF1/MAFbx/FOXO pathways and potential triggers behind muscle disuse, there is a significant gap in knowledge in the regulatory mechanism of the associated genes and their functional significance. It is known that anabolic and catabolic pathways regulate muscle atrophy in adult organisms. Deacetylase inhibitors represent a prototype of epigenetic drugs that have been proposed as a possible intervention that targets multiple signaling pathways in the pathogenesis of muscle atrophy. Niclosamide has also been proposed to regulate myogenesis and catabolic pathways in skeletal muscle.

Apart from microgravity, radiation exposure in spaceflight has been reported to aggravate atrophic processes in soleus and gastrocnemius muscles, which is induced already by spaceflight. Radiation was shown to inhibit the reparative processes [9]. Oxidative stress is increased by higher levels of radiation. The upregulation of heme oxygenase-1 (HO-1) counters cellular damage due to radiation which can be artificially induced [10]. Several countermeasures have been proposed for alleviating muscle wastage in spaceflight. Exercise countermeasures do not alleviate the reduction in muscle function or muscle size due to the unloading effects of spaceflight [11]. While exercise countermeasures seem insufficient for maintaining muscle function in long deep space measures, it is important to find effective countermeasures for long duration spaceflights. Bone loss is preserved and tibialis anterior and gastrocnemius muscle changes are eliminated by countermeasures such as bisphosphonates and anti-RANKL therapies (Denosaumab and OPG-Fc) and treatment of young mice with REGN1033 (a monoclonal antibody against myostatin) [12]. With future space missions, finding effective countermeasures for muscle atrophy in spaceflight has gained paramount importance. Simulated microgravity, use of animal models, applications of countermeasures, studies of interrelationships between bone and muscle tissues, and studies on the effect of radiation on skeletal muscles are necessary for human exploration of space [13]. In our earlier paper on drug repurposing [14], we applied three Machine Learning (ML) methods for identifying drugs for treatment of organ muscle atrophy. In this paper, we have added the Pearson correlation method for identification of key gene regulators of skeletal muscle atrophy, and also have implemented Graph Convolutional Neural Network (GCN) for link prediction. GCN results for identification of repurposable drugs for skeletal muscle atrophy is compared with the GNN method reported as the best method in [14]. NASA’s GeneLab [15] datasets are collected in spaceflight under microgravity and low radiations doses in low Earth orbit. The radiation details of these datasets are provided in [16]. Section 2 presents the GeneLab datasets and ML methods used to identify key diseases associated with skeletal muscle atrophy and drugs for repurposing. Section 3 presents the results of the ML algorithms for link predictions in the constructed Gene Disease Knowledge Graph (GDKG) and Disease Drug Knowledge Graph (DDKG). Section 4 discusses the key genes and repurposable drugs selected by link prediction, and Section 5 presents the conclusions.

## 2. Materials and Methods

Datasets from the GeneLab repository [15] related to skeletal muscle atrophy were mined for studying the effects of microgravity and low radiation doses in low Earth orbit found beyond Earth’s atmosphere on mice. All the -omics datasets in GeneLab were preprocessed and normalized before being published.

### 2.1. GeneLab Datasets

GLDS-99, 101, 103, 104: A cohort of 16-week-old female mice were flown in the ISS for 37 days. They were euthanized in spaceflight and returned to Earth where left and right extensor digitorum longus muscle tissue (GLDS-99), gastrocnemius (GLDS-101), quadriceps (GLDS-103), and left and right soleus muscles (GLDS-104) samples were collected. RNA and DNA sequencing was carried out. GeneLab processed the RNA sequencing data into gene expression values using standardized methods. These datasets belong to the Rodent Research (RR) payload. The daily average absorbed dose of Galactic Cosmic Radiation (GCR) nucleic particle is 0.13126 mGy, Inner Radiation Belt (IRB) South Atlantic Anomaly (SAA) is 0.07331 mGy, and the cumulative absorbed dose of GCR is 4.98795 mGy, and SAA is 2.78573 mGy.

GLDS-111 and GLDS-135: Adult male mice C57BL/N6 were flown aboard the BION-M1 biosatellite for 30 days on orbit (BF) or housed in a replicate flight habitat on Earth (BG) as the reference flight control. GeneLab processed RNA sequencing data from mouse soleus and EDL muscles (GLDS-111) and longissimus dorsi and tongue (GLDS-135). The radiation inside the Bion-M1 mouse habitat dosimeters SPD2 and SPD4 recorded an average absorbed dose of 0.630 and 1.149 mGy, respectively. These are averages of low and high LET radiation doses. The total average absorbed radiation dose for the mission is 18.81 mGy and 34.30 mGy for the SPD2 and SPD4 dosimeters, respectively. The total average absorbed dose of Galactic Cosmic Radiation (GCR), Outer Radiation Belt (ORB), and Inner Radiation Belt (IRB) is 0.985 mGy.

GLDS-21: Mice were flown on the STS-18 shuttle flight mission for 11 days, 19 h and gene expression analysis was performed on gastrocnemius muscle. Mice were maintained on earth for the same period. Additionally, to identify changes that were due to unloading and reloading, ground-based mice were subjected to hind limb suspension for 12 days and microarray analyses were conducted on their calf muscle. The average absorbed radiation dose is 2.19 mGy for the entire mission with an average absorbed radiation dose rate of 0.18 mGy.

**Figure 1:**
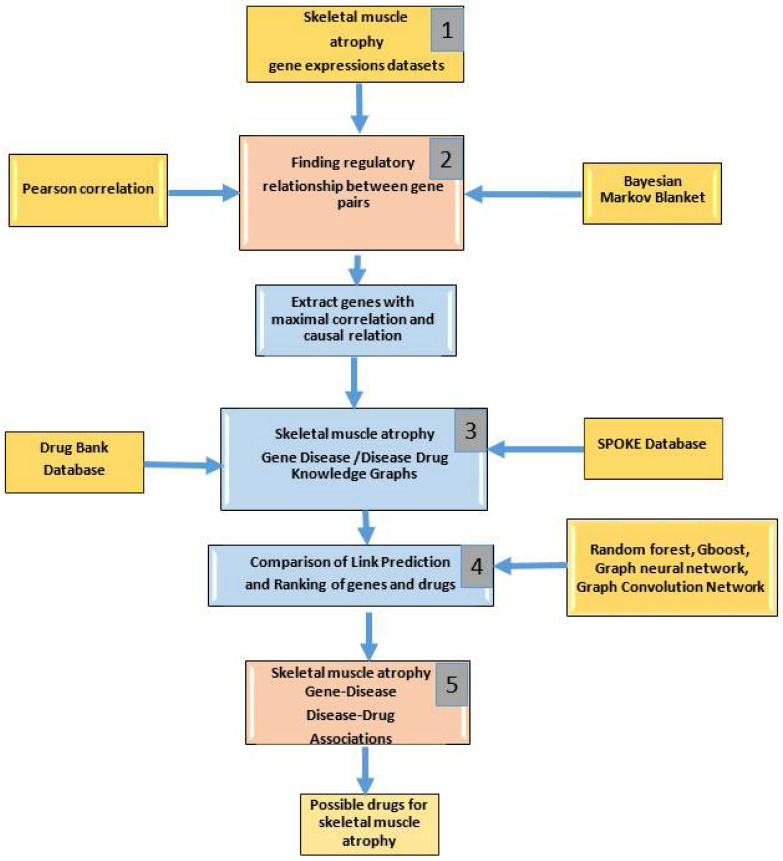
Workflow pipeline showing the order of steps involved in constructing GDKG and DDKG and link prediction methods for finding key diseases associated with skeletal muscle atrophy genes and drugs for repurposing.

The workflow pipeline for identifying key genes and drugs for treating skeletal muscle atrophy is shown in Figure 1. The stages of the pipeline are numbered from 1 to 4 and each stage is explained below.

### 2.2. Finding Regulatory Relationships between Gene Pairs (Stage 1)

Graph-based Gene Regulatory Network (GRN) inferencing methods of Pearson correlation and Markov Blanket (MB) are utilized to identify the most regulated genes in the seven GeneLab datasets [17,18]. The gene expression values of pairs of genes are used to compute the Pearson correlation value. The p-values are used to extract the most correlated pairs of genes by selecting all values below 5 × 10^−7^, which will extract the same pairs of genes as a correlation threshold of 0.9 and above. For identifying causal relational gene pairs, the Markov Blanket (MB) method is used. Joint conditional probabilities are computed from the gene expression values which are used to construct a Bayesian Network (BN). The incremental association Markov blanket of any node (gene) in a BN is the set of parents, children, and spouses (the other parents of their common children) of the gene. The genes are connected by edges if its upregulation is caused by another gene, or if it causes the upregulation of another gene. The MB(X) of a node (gene) X includes its parents, children, and spouses which are the strongly relevant genes to gene X. The output is a list of pairs of genes that are connected by edges. The list of pairs of most correlated genes and causally related genes are combined into one list and input to the next stage in Figure 1.

### 2.3. Construction of Knowledge Graphs (Stage 2)

The selected genes from Stage 1 are input to the Scalable Precision Medicine Open Knowledge Engine (SPOKE), which is a database of databases [19]. SPOKE is used for creating a network based on a data integration approach to prioritize disease-associated genes [20]. It is a graph-theoretic database organized in a hierarchical manner with inputs from molecular research, clinical insights, environmental data and others. Currently it integrates 19 different databases. The SPOKE creates a new graph with the provided list of skeletal muscle atrophy genes and the diseases associated with it. The list of genes and their associated diseases are input to Cytoscape to construct the Gene Disease Knowledge Graph (GDKG). The Disease Drug Knowledge Graph (DDKG) is constructed by finding the top ten drugs used to treat the diseases associated with skeletal muscle atrophy from the DrugBank database. A table of diseases and the top ranked drugs is built and input to Cytoscape to construct the DDKG.

#### Graph Concepts and Properties for Analysis of GDKG and DDKG

Graph concepts of random walk and preferential attachment used by the link prediction algorithms are described in this section. We also compute network measures on the constructed graphs. We follow Janwa, Massey, Velev and Mishra [21,22,23,24]. A graph is a representation of a set of entities and relations among them and represents an underlying concrete network, such as a GRN, the internet, or a social network. We formally present a graph as a pair of sets G=(V, E), where V are the vertices (nodes, points) and E⊆V×V are the edges (arcs), respectively. When E is a set of unordered pairs of vertices, the graph is said to be undirected. In a directed graph (representing key genes and target genes, for example) G=(V, E, o, t), E consists of an ordered set of vertex pairs, i.e., for each edge e∈E, e→(o(e), t(e)) where o(e) is called the origin of the edge e and t(e) is called the terminus of the edge e [22,23]. A graph is weighted if there is a map (weighting function, $w:E\to {\mathbb{R}}_{+}$), assigning to each edge a positive real-valued weight. Weighting can represent the strength of a signal in sender–receiver gene interaction, for example. A network’s properties are governed by its topology, such as the degree distribution, clustering coefficients, motifs, assortativity, hierarchicity, etc. (see [24,25,26]); a more in-depth treatment regarding biomedical networks is given in [27]. The degree of a vertex v, deg(v), is the number of edges that connect the vertex with other vertices. In other words, the degree is the number of immediate neighbors of a vertex. In directed graphs, the in-degree and out-degree of a vertex can be defined as the number of incoming and outgoing edges, respectively. Thus, the degree distributions can tell a great deal about the structure of a family of networks. As probability distribution, degree distribution can be binomial, Poisson, or Gaussian (in the limit), or as we will see, it can follow a power–law distribution that is characterized by a scale-free property. We say that a graph is *sparse* if ⟨k⟩=O(logN) (or M=O(N logN)). In random probability models such as the Erdos-Renyi model, one does not find nodes of a very high degree.

Similarity measures computed from neighborhoods in a graph are widely used in link prediction algorithms [28]. A semi-supervised scalable feature learning method is proposed in [29], where the authors develop a family of biased random walks resulting in a flexible search space of nodes for link (edge) prediction. We have used this method to obtain the highest ranked nodes for possible links between the muscle atrophy gene and its disease associations, as well as between diseases and drugs in the Graph Neural Network (GNN) method.

Random walks: A walk of length n in a graph is a sequence of alternating vertices and edges, $\left\langle v_{0},\;e_{1},\;v_{1},\;e_{2},\;\ldots ,\;e_{n},\;v_{n}\right\rangle$ such that $0\left(e_{i}\right)=v_{i-1}$ and $t\left(e_{i}\right)=v_{i}$ for all i=1, …,n. Let T be the diagonal matrix with $d_{v}$ along the diagonal. First, we consider the stochastic matrix $P=T^{-1}A$, which may be thought of as describing the probabilities of certain “information” being moved from one node to a neighboring node by a diffusion process. Let $\left\{v_{0},e_{1},v_{1},e_{2},\;\cdots v_{s}\right\}$ be a random walk in the graph with $\left(v_{i-1},\;v_{i}\right)\in E\left(G\right)$, for all 1≤i≤s, and determined by transition probabilities $P\left(u,v\right)=Prob(x_{i+1}=v\;|x_{i}=u)$ which are independent of i. Normally, we take $p\left(u,v\right)=w\left(u,v\right)/d_{u}$, as defined by the stochastic matrix P. Apart from random walks, we have computed preferential attachment measures to obtain possible gene–disease and disease–drug link associations. We follow [30] for computation of preferential attachment. For any node u let Γ(u) denote the set of neighbors of u. Let Λ be a community of G, i.e., Λ is a set of cohesive vertices such that it contains more connections inside the set than outside the set. The preferential attachment score of u and v is defined as |Γ(u)||Γ(v)|.

### 2.4. ML Methods for Link Prediction (Stage 3)

We used four ML methods for identifying and ranking the top skeletal muscle gene disease associations in the GDKG, and for identifying the top ranked drugs for repurposing from DDKG. The Random Forest (RF), Gradient Boost (GB), and Graph Neural Network (GNN) were used for link prediction and drug repurposing for organ muscle atrophy [14]. In addition to the above, we implemented the GCN method. The problem of link prediction is to predict an edge between two existing nodes in a graph or network. Each of the methods are described below.

#### 2.4.1. Random Forest (RF) Method

This method is based on decision trees, and an ensemble of trees is called a decision forest. Each tree is trained on a random subset of input features, and their predictions are combined to improve overall prediction. The tree is based on discriminants instead of likelihoods. Discriminants are estimated by passing class densities. The hyperparameters area: tree depth of 15 with 500 estimators.

#### 2.4.2. Gradient Boosting (GB) Method

The GB method is also an ensemble decision tree method which trains one tree at a time. The regression trees were built from the previous step on the prediction error of the previous tree. This is a useful method for tabular datasets. Multiple weak learners are combined to give a better performance. It can find nonlinear relationships between model targets and features and can deal with outliers, and missing values. The feature labels are the value of various node centralities. The positive and negative samples are the labels for the existent and non-existent edge in the network, respectively. The features of the nodes at the end of the edges, along with the positive or negative label, form a well-defined dataset for the task of link prediction. The learning rate is 0.2 for this algorithm.

#### 2.4.3. Graph Neural Network (GNN) Method

The GNN is a deep network with ten hidden layers with 100 nodes (neurons) in each of the hidden layers. The activation function for the hidden layers is the Rectified Linear Unit (ReLu) function. The limited-memory Broyden–Fletcher–Goldfarb–Shanno (lbfgs) solver from sktlearn library in Python was used to predict the links. The input layer of the GNN takes as input random walk features computed on the knowledge graphs. The output of the GNN is a matrix of predicted edges.

#### 2.4.4. Graph Convolution Neural Network (GCN)

We used the Graph Convolution Neural Network (GCN) for link prediction in GDKG and DDKG for skeletal muscle atrophy and compared it with the above methods. The GCN takes as input the knowledge graph with N number of nodes, A is the N×N adjacency matrix. The GCN learns the graph $G_{i}=\left(V_{i},\;E_{i}\right)$, learns node embeddings, and predicts links between the nodes. The layer-wise propagation rule for each neural network layer is

$$H^{\left(l+1\right)}={\bar{D}}^{-\frac{1}{2}}\bar{A}{\bar{D}}^{-\frac{1}{2}}\;H^{\left(l\right)}W^{\left(l\right)}.$$

**Figure 2:**
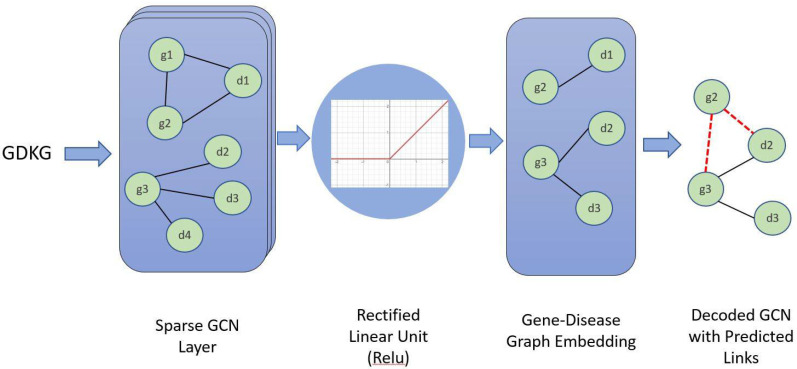
Graph Convolutional Network (GCN) is trained on the GDKG network. Figure shows the sparse GCN layer, ReLu activation function, graph embedding, and the decoded GCN with output predicted links between the genes and disease nodes. g1 and g2 are the gene nodes, d1, d2, and d3 are disease nodes. The output predicted links are shown as red dotted lines.

Here, $\bar{A}=A+I_{N}$ is the adjacency matrix of the undirected graph G with added self-connections. $I_{N}$ is the identity matrix, $D_{ii}=\underset{j}{\sum }\bar{A_{ij}}\;$ is the diagonal node degree matrix of A and $W^{\left(l\right)}$ is the layer-specific trainable weight matrix, σ(.) is an activation function. With spectral analysis, a graph convolution is a multiplication of spectra of signal in a Fourier domain [31]. As it is computationally expensive, the convolution kernel is the existing Chebyshev polynomial of Eigenvalues in a spectral domain. A softmax activation function is applied row-wise to f(X,A) to obtain $Z=soft\;\underset{\;}{\max}f\left(X,A\right)$ where $soft\;\underset{\;}{\max}(x_{i})=\frac{\exp\left(x_{i}\right)}{\sum_{i}x_{i}}$. To evaluate loss in this semi-supervised model, cross-entropy error is calculated as follows: $L=-\overset{\;}{\underset{l\in y_{L}}{\sum }}\overset{F}{\underset{f=1}{\sum }}Y_{lf}\ln Z_{lf}$ where $Y_{L}$ is the set of nodes with labels or the labeled training instances. The weights of the neural network W are trained using gradient descent. Figure 2 shows the GCN trained for link prediction on the GDKG. The GCN has two hidden layers with 32 nodes in the first hidden layer and 16 nodes in the second hidden layer, respectively. The GCN uses Adam optimizer for gradient descent and weight updates for the network. The probabilities of the predicted links range from 0 to 1. These probabilities are predicted using the ReLu activation function shown in Figure 2.

### 2.5. Gene-Disease and Disease-Drug Associations (Stage 4)

The knowledge graphs are split into training and validation sets. The GridSearchCV library is used to estimate the best split of the data for cross validation. This implementation uses 10-fold cross validation for link prediction in both the knowledge graphs. The computation of network features and graph features are implemented in Python using the libraries networkX, node2vec, pandas, numpy, and sktlearn. The link prediction accuracies for the four methods are calculated by comparing a binary label (an edge exists or not exists) with a real valued predicted score. The technique used for evaluation in this setting is the Area Under the Receiver Operating Characteristic (AUROC) curve. The predicted links are sorted from highest probability to lowest probability. The drug nodes with the highest link probability to the disease nodes are selected as candidates for repurposing.

## 3. Results

The seven gene expression datasets have from three to eight expression values. The datasets were combined, and the significantly regulated genes were extracted using the Pearson correlation and Incremental Association Markov Blanket (IAMB) methods. For details on the implementation of Pearson correlation and IAMB, please refer to [32]. Pearson identified the most correlated genes and IAMB identified causally related genes. A total of 473 genes were identified as the most significantly regulated from the seven datasets. Hence, we have included all of these genes in our analysis as important regulators of skeletal muscle atrophy in spaceflight.

**Figure 3:**
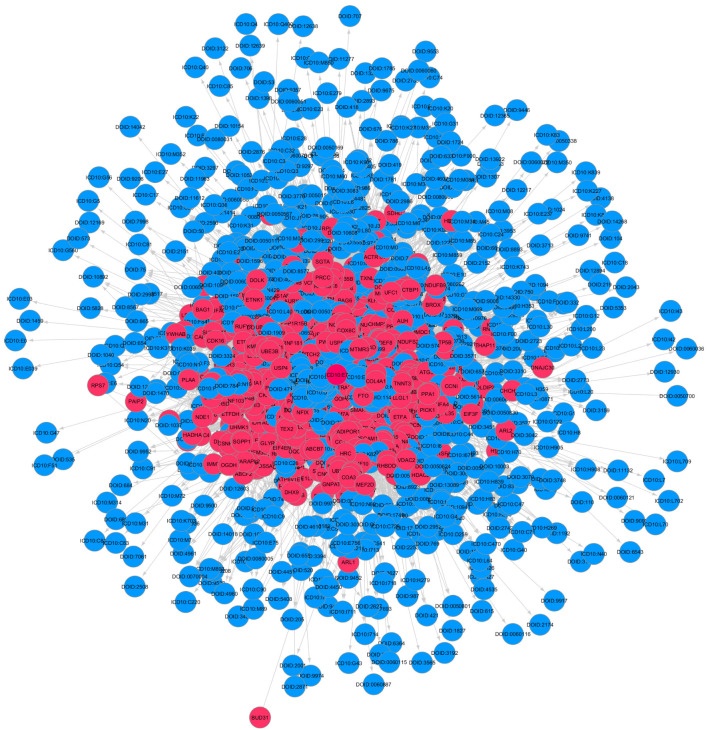
Gene Disease Network (Red nodes–Genes, Blue nodes–Disease).

Many diseases such as metabolic and neuromuscular diseases, cancer, chronic inflammatory diseases, and acute critical illness are associated with skeletal muscle atrophy, muscle weakness, and general muscle fatigue. Additionally, skeletal muscle atrophy is the secondary effect of many diseases, and it is important to find the diseases linked with this condition. The Scalable Precision Medicine Knowledge Engine (SPOKE) was used for identifying all the diseases related to muscle atrophy. SPOKE is a large heterogeneous network with many types of biological data organized in a hierarchical structure for the benefit of biomedicine and human health (Scalable Precision Medicine Knowledge Engine n.d.). The maximally regulated genes identified from the GRNs were input to the SPOKE. Figure 3 shows the GDKG constructed from all the diseases related to the muscle atrophy genes. Next, we applied ML methods to predict new gene disease associations in the GDKG.

### Link Prediction Using GCN and Other ML Methods

**Figure 4:**
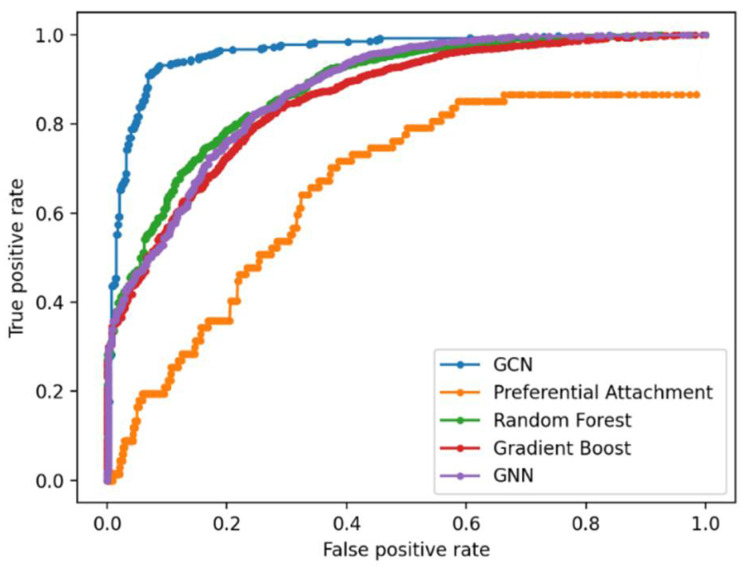
Receiver Operating Characteristic (ROC) curve showing true positive and false positive scores for link prediction in the GDKG using the five methods.

**Figure 5:**
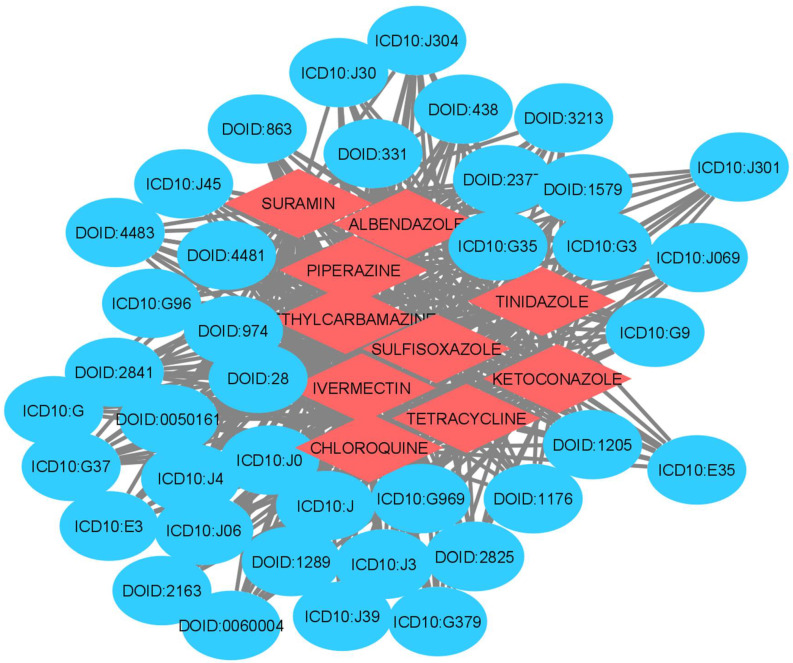

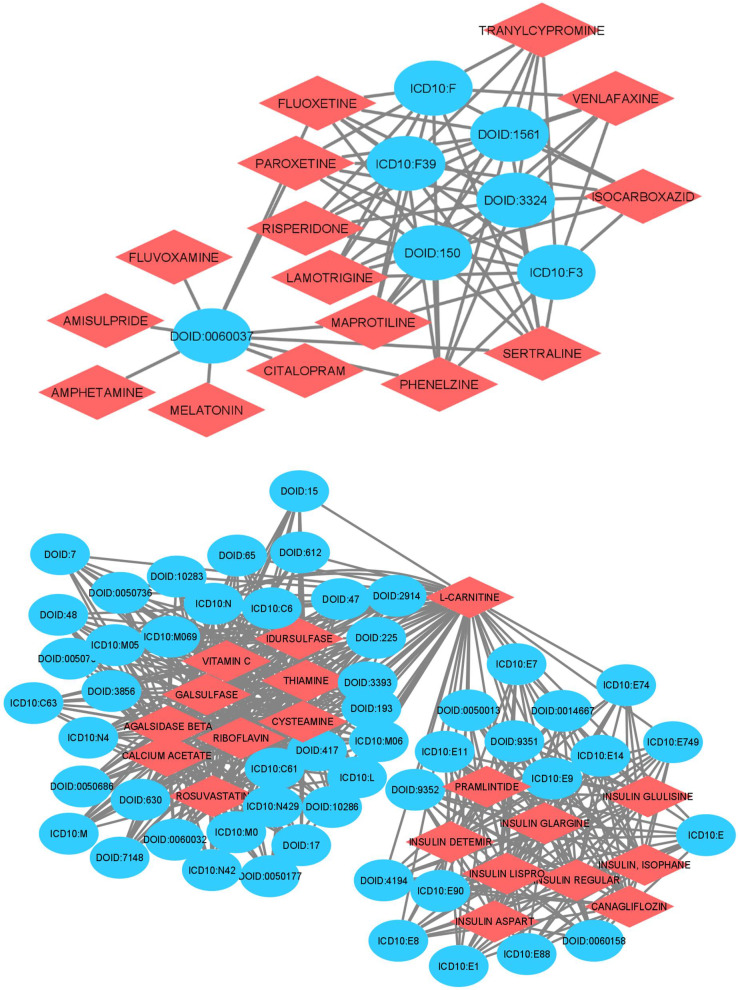

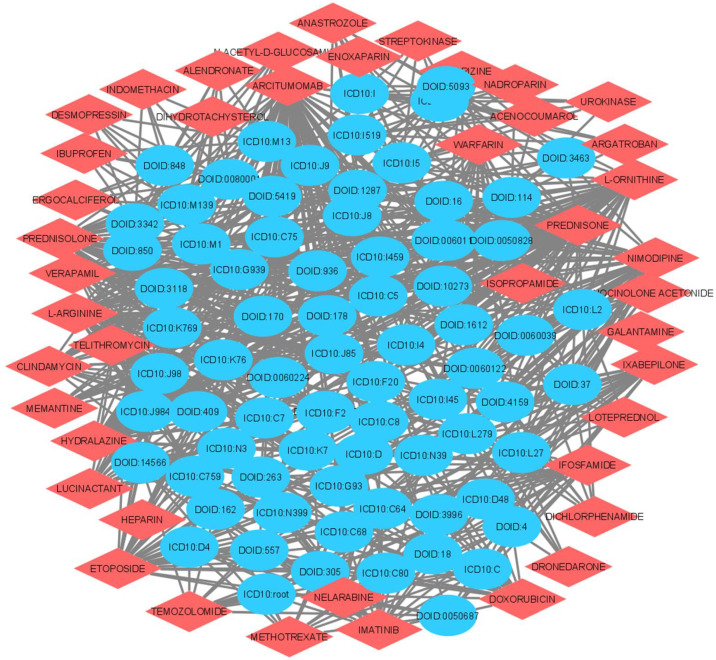
Disease Drug Network (Red nodes—Drugs, Blue nodes—Diseases).

**Figure 6:**
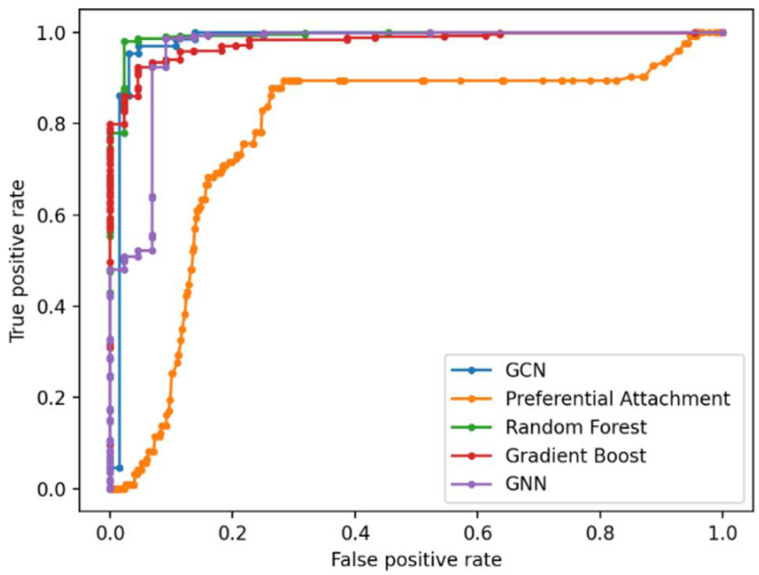
Receiver Operating Characteristic (ROC) curve showing the true positive and false positive scores for link prediction in the DDKG using the five methods.

The graphs were preprocessed by computing the graph Laplacian. Each node was embedded into a feature vector and input to two hidden layers. Given the graph embedding, GCN model is trained to predict new gene–disease interactions in the GDKG. The GCN predicted 21 new gene disease associations with a probability greater than 0.8. The gene names and associated diseases are given in Table 1. Figure 4 shows the Receiver Operating Characteristics (ROC) curve for link prediction using the GCN and GNN, Random Forest, Gradient Boosting, and preferential attachment methods. The link prediction methods were trained with 80% of the data and the remaining 20% were used for testing. The ten-fold cross validation accuracies for the gene-disease link prediction using the four methods are given in Table 2. The key diseases associated with skeletal muscle atrophy genes were identified and sorted. Out of these top ranked, 100 diseases were selected. The drugs were selected from the drug bank database [33] and the ten most commonly used drugs for each of the diseases were selected. The Disease–Drug Knowledge Graph (DDKG) was then built from the diseases and drugs used to treat them. The DDKG is shown in Figure 5. Since the existing drugs are the most commonly used for these diseases, the link prediction method was used to find new repurposable drugs for these diseases which in turn can be used for repurposing for muscle atrophy in spaceflight. Figure 6 shows the Receiver Operating Characteristics (ROC) curve for link prediction using the GCN, GNN, Random Forest, Gradient Boosting, and preferential attachment methods applied to the DDKG. A total of 60% of the data from the DDKG was used for training and the remaining 40% for testing. Table 3 lists the new predicted links with the highest probabilities for disease and drugs using the GCN link prediction method. The predicted links with highest probabilities for drugs and diseases using the GNN method is given in Table 4 for comparison. The ten-fold cross validation accuracies for link prediction applied to DDKG are given in Table 5. The GDKG and DDKG are massively scalable knowledge graphs and have several properties, such as expansion and diffusion. Graph network measures computed on these graphs are listed in Table 6. The preferential attachment network measure-based link prediction gives an accuracy of 74.64% for the GDKG and 73.55% for the DDKG, respectively.

We have compared the GCN-based link prediction in the knowledge graphs with other ML methods, Random Forest, Gradient boosting, GNN, and preferential attachment. The GCN method demonstrated the best performance with highest accuracies from ten-fold cross validation for link prediction in both the GDKG and DDKG.

## 4. Discussion

All of the 423 genes in the GDKG are highly activated and related to muscle atrophy in spaceflight. However, it is necessary to identify a few most important genes related to other conditions that can enable the identification of drugs for repurposing. The GCN link prediction method has achieved the highest accuracy of 96.11%, as seen from AUROC values for the ten-fold cross validation accuracies for the four methods of RF, GB, GNN and GCN given in Table 2. The GCN link prediction method has predicted 20 important genes. Their association with other diseases [34] is given in Table 1. For example, *RPS25* is an mRNA significantly affected in spaceflight gastrocnemius [5] and its reduction in bed rest [35]. From Table 1, we see that this gene is not only significantly activated in muscle atrophy but is also associated with disorder of central nervous system. Similarly, many of the muscle atrophy genes in Table 1 such as *SNF8* [36], *ELK4* [37], *FTO*, and *EIF3H* are associated with neurodegenerative diseases. The Eukaryotic Initiation Factor (*EIF*) is one of the most complex translation initiation factors and consists of several subunits. The EIF3 complexes are central regulators of atrophy in skeletal muscle and are also linked to neurodegenerative diseases [38]. Muscle activity causes the ubiquitin-proteasome system to remove sarcomeric proteins. A decrease in muscle mass is associated with: (1) increased conjugation of ubiquitin to muscle proteins; (2) increased proteasomal ATP-dependent activity; (3) increased protein breakdown that can be efficiently blocked by proteasome inhibitors; and (4) upregulation of transcripts encoding ubiquitin, some ubiquitin-conjugating enzymes (E2), a few ubiquitin-protein ligases (E3) and several proteasome subunits [39]. The proteins such as *NDUFS3* identified by the GCN link prediction methods are important for reversion of myopathies in mice [40]. These are atrophy associated proteins (*NDUFS3*, *NDUBF2* part of the ubiquitin-proteasome system [41]. The loss of other target genes such as *MEF2A* results in progressive atrophy [42]. Myostatin, a member of the TGF-ꞵ family is a negative regulator whose predominant secretion in skeletal muscles causes muscle atrophy. Similarly, an increase in autophagy related gene ATG3 is identified by GCN link prediction [43]. Resistive Exercise (RE) with superimposed vibration mechanosignals (RVE) is proposed to counter muscle atrophy, which is effective against the over expression of Mitochondrial Ribosomal Proteins (MRPs) and Mitochondrial Tu Translation Elongation Factor (*TUFM*) that cause muscle atrophy [44]. Some of the MRP proteins are identified to be linked with other diseases such as cancer. Lack of Zinc Finger RNA-binding (*ZFR*) proteins also cause severe muscle wasting [45]. The collagen β(1-O)galactosyltransferase type 1 (*COLGALT1*) has been identified, whose loss of function also causes muscle atrophy [46]. Many proteins such as *RPL7A* have increased expression in cancer [47]. Other critical regulators of muscle atrophy such as protein arginine methyltransferases (*PRMTs*) -*PRTM5* is linked by the GCN method [48]. Other genes such as *SNW1* are also prioritized in other diseases such as Amyotrophic Lateral Sclerosis (ALS) [49]. Hence, we find that genes overexpressed in skeletal muscle atrophy are also found to be prioritized in other diseases such as cancer, and neurodegenerative diseases. Mitochondria-related gene *MRPS21* has been identified here as well, whose declined expression has been found in sarcopenia or age-related skeletal muscle deterioration [50].

The four ML link prediction methods are applied to the DDKG. As seen from Table 5, the GCN method obtains the highest accuracy of 99.19%. The top ranked drugs with new predicted links and highest probabilities above 0.7 using the GCN method are listed in Table 3. The drug L-carnitine is an essential nutrient that has been proposed as a dietary supplement to enhance ꞵ-oxidation and treat skeletal muscle atrophy conditions [51]. This nutrient is predicted with the highest probability by the GCN method. This is followed by thiamine, which is also an essential nutrient that has been selected by the GCN method. Thiamine is another nutrient whose deficiency causes myotonic dystrophy. It has been found that treating patients with intramuscular thiamine 100mg twice a week for 11 to 12 months is effective in improving muscle strength [52]. Both L-carnitine and thiamine are potential nutrients that can be given as a dietary supplement countermeasure for skeletal muscle atrophy in spaceflight. There is no specific treatment for muscle atrophy, with only recent advances in the identification of treatments such as nanotechnology approaches [53]. However, ML based methods such as the GCN can be used to select drugs. The drugs selected by the GCN method for repurposing are commonly used for the treatment of diseases that are associated with skeletal muscle atrophy. Bimagrumab is an anabolic medication used for treating muscle wasting in COPD [54]. Arcitumomab and golimumab are drugs belonging to the Monoclonal AntiBodies (MABs) family predicted by the GCN method (Table 3). Decline in anabolic signals and activation of catabolic pathways contribute differently to muscle atrophy pathogenesis associated with diseases or unfavorable conditions such as spaceflight. Hence, epigenetic drugs have been proposed [55] to target multiple pathways. Fluocinolone acetonide is a corticosteroid with glucocorticoid activity selected by the GCN method, which could be a useful drug for repurposing for skeletal muscle atrophy. As mentioned in [56], niclosamide is not a good drug for repurposing for glucocorticoid-induced muscle atrophy or cancer cachexia. Anti-inflammatory drugs such as dexamethasone, and drugs alendronate have been proposed for the therapeutic management of muscle wasting and sarcopenia [57]. Similar drugs such as hydrocortisone and chloroquine are selected by link prediction. Insulin resistance is a significant cause of decreased protein and glucose available for muscle anabolism [58]. It can be noted from Table 3 that four insulin related medications have been selected for repurposing. The four drugs: L-carnitine, clindamycin, vitamin C, L-ornithine, and nelarabine selected by GCN, have also been selected by the GNN method with new predicted links and higher probability as seen in Table 4. Additionally, the common top ranked diseases with predicted links using GCN and GNN from the DDKG are metabolic diseases, type 2 diabetes, cancer, and neurological disorders. Although there is some overlap in the identified diseases and drugs using the GCN and GNN methods, the drugs predicted by the GCN method are more reliable, as this method has the highest accuracy for the link prediction probabilities. It has better performance in training with lesser samples, and validation accuracies.

The graph-theoretic measures of degree distribution, neighborhood connectivity, Eigenvector centrality, and subgraph centrality for the nodes in the GDKG and DDKG are listed in Supplementary Table S1 for the 473 genes, and in Supplementary Table S2 for the 98 drugs, respectively. The degree distribution ranges from 1 to 171 for the gene nodes in the GDKG network and between 5 to 76 for the drug nodes in the DDKG network, respectively. Some of the gene nodes, as well as drug nodes, have a higher number of connections in the networks. The neighborhood connectivity is higher in the GDKG because the network is constructed using a large number of diseases overlapping with skeletal muscle atrophy. The neighborhood connectivity is ten for all the drug nodes in the DDKG because we selected a maximum of ten significant drugs for each disease. The Eigenvector centrality is a measure of the influence of a node in a network, the higher this score, the greater the connectivity of this node with nodes that have a higher score for the same measure. This measure is similar for the genes and the drugs in both networks. The subgraph centrality of a node is a weighted sum of the numbers of all closed walks of different lengths in the network starting and ending at the node. There are more closed walks for the gene nodes in the GDKG, hence this value is higher for the gene nodes in GDKG than the drug nodes in the DDKG. The graph theoretic measures for the whole GDKG and DDKG networks are given in Table 6. The DDKG network has a higher value of spectral gap, indicating that the network is sparse, and has higher measures for random walk, diffusion, and expansion. The GDKG network has a higher average number of neighbors, indicating that the skeletal muscle genes have higher neighborhood connectivity measure.

The preferential attachment network measure-based link prediction gives an average accuracy of 74.10%, while the ML-based methods give accuracies above 80%. The random walk measure is shown to be a better network measure for link prediction than preferential attachment. The ML methods of GNN, RF and GB which use random walk features perform better than preferential attachment-based link prediction alone. The ML method of GCN that uses semi-supervised learning of the graph structure by node embeddings performs best for link prediction in both the GDKG and DDKG networks giving an accuracy of 96.11% and 99.19% in the GDKG and DDKG networks, respectively. The average accuracy of the GNN, RF, and Gboost method for link prediction in the GDKG network is 88.69%, whereas the GCN gives a much better accuracy of 96.11%. Overall, ML methods can be used for novel applications such as the identification of new gene regulators of diseases from spaceflight datasets and candidate drugs for their treatment.

## 5. Conclusions

Though skeletal muscle atrophy is known to be an incapacitating consequence of several chronic diseases, increasing morbidity and mortality, no drug is approved to treat this condition. It also severely affects animal models flown in spaceflight missions. In this paper, we have presented a comprehensive study on skeletal muscle atrophy identifying the key genes that give rise to this condition in spaceflight microgravity. By the application of ML algorithms, we have identified the main gene regulators of skeletal muscle atrophy that are also highly activated in other diseases. By constructing disease drug networks and applying ML algorithms for link prediction, we have identified top ranking drugs with the highest probability that are novel candidates for the management of skeletal muscle atrophy in spaceflight microgravity. In this work, we have mined seven GeneLab datasets to identify key genes and drugs. Through network analysis and ML methods, we show that our networks are scalable and can be expanded to include as many datasets, genes and drugs for speeding up the process of identifying repurposable drugs for medical conditions that arise in long duration spaceflights.

## Figures and Tables

**Table 1:** Ranking of genes and diseases with new predicted links using GCN.

Gene	Disease Code	Link Prediction Probabilities	Disease Name
*EIF3H*	ICD10:C22	0.92	Malignant neoplasm of liver and intrahepatic bile ducts
*SNF8*	DOID:178	0.83	vascular disease
*RPS25*	ICD10:G969	0.77	Disorder of central nervous system
*NDUFB2*	DOID:0050589	0.79	inflammatory bowel disease
*MTCH2*	DOID:10273	0.95	heart conduction disease
*FTO*	DOID:1289	0.79	neurodegenerative disease
*NDUFS3*	ICD10:I5	0.97	Non-ischemic myocardial injury (non-traumatic)
*MEF2A*	ICD10:C25	0.79	Malignant neoplasm of the pancreas
*DDA1*	DOID:8857	0.84	lupus erythematosus
*ATG3*	ICD10:H8	0.8	disorder of vestibular function
*COG6*	ICD10:N429	0.73	Disorder of prostate
*ELK4*	DOID:6364	0.71	migraine
*MRPL4*	DOID:2007	0.73	Pesticide residues in food
*ZFR*	ICD10:N399	0.78	Disorder of urinary system
*ELK4*	ICD10:G93	0.89	brain disorder
*COLGALT1*	DOID:0050890	0.84	synucleinopathy
*RPL7A*	ICD10:K0	0.81	Diseases of the oral cavity and salivary glands
*PRMT5*	ICD10:N399	0.86	Disorder of urinary system
*MRPS21*	DOID:0050687	0.89	cell type cancer
*SNW1*	ICD10:C64	0.83	Malignant neoplasm of kidney

**Table 2:** Ten-fold cross validation accuracies for link prediction using RF, Gboost, and GNN in GDKG.

Methods	1	2	3	4	5	6	7	8	9	10	AUROC
RF	89.39	88.64	90.54	91.76	89.09	88.57	86.04	91.03	87.66	90.94	88.75
GB	85.28	84.62	87.03	86.82	85.62	86.52	83.22	87.33	82.89	88.06	85.69
GNN	87.70	90.13	89.78	89.96	88.79	90.59	85.60	89.44	88.35	90.26	88.63
GCN	88.95	90.87	91.21	92.79	93.00	93.20	94.29	95.05	95.72	96.00	96.11

**Table 3:** Ranking of drugs and diseases with new predicted links using GCN.

Drugs	Disease	Link Prediction Probability
L-CARNITINE	Metabolic disease	1
THIAMINE	Autoimmune disease of the musculoskeletal system	1
TELITHROMYCIN	Breast cancer	0.98
FLUOCINOLONE ACETONIDE	Uterine disease	0.96
RIBOFLAVIN	Autoimmune disease of the musculoskeletal system	0.94
AZATHIOPRINE	Cardiovascular system disease	0.94
IVERMECTIN	Allergic rhinitis	0.9
INSULIN LISPRO	Urinary system disease	0.9
NELARABINE	Hypervitaminosis	0.9
SURAMIN	Allergic rhinitis	0.89
TETRACYCLINE	Male reproductive organ cancer	0.86
INSULIN DETEMIR	Urinary system disease	0.85
PRAMLINTIDE	Type 2 diabetes mellitus	0.84
ARCITUMOMAB	Breast cancer	0.83
CLINDAMYCIN	Influenza and pneumonia	0.83
L-ORNITHINE	Vasomotor and allergic rhinitis	0.83
BUDESONIDE	Autoimmune thyroiditis	0.82
GOLIMUMAB	Benign neoplasm	0.82
ARCITUMOMAB	Skin disease	0.82
INSULIN, ISOPHANE	Unspecified diabetes mellitus	0.82
HYDROCORTISONE	Integumentary system cancer	0.82
CHLOROQUINE	Bone inflammation disease	0.82
L-CARNITINE	Malignant neoplasm	0.82
INSULIN GLARGINE	Disease of the genitourinary system	0.81
KETOCONAZOLE	Allergic rhinitis	0.8
WARFARIN	Generalized skin eruption	0.79
ARCITUMOMAB	Nasal cavity disease	0.79
KETOCONAZOLE	Malignant neoplasm of prostate	0.79
VITAMIN C	Lung disease	0.78
GALSULFASE	Malignant neoplasm of other endocrine glands	0.77
L-ORNITHINE	Arterial fibrillation	0.75
LUCINACTANT	Mood disorder	0.75
VITAMIN C	Mental, behavioral and neurodevelopmental disorders	0.74
TETRACYCLINE	Allergic rhinitis	0.74
SURAMIN	Other disorders of central nervous system	0.73
SULFASALAZINE	Other and unspecified noninfective gastroenteritis and colitis	0.71
TINIDAZOLE	Bronchial disease	0.71

**Table 4:** Ranking of drugs and diseases with new predicted links using GNN.

Drugs.	Disease Name	Link Prediction Probability
MEMANTINE	Carcinoma	0.98
CINNARIZINE	Carcinoma	0.97
MEMANTINE	Heart Disease	0.97
IXABEPILONE	Complications Additionally, Ill-Defined Descriptions Of Heart Disease	0.96
PREDNISOLONE	Malignant Neoplasm of Other Additionally, Unspecified Urinary Organs	0.95
CLINDAMYCIN	Artery Disease	0.93
CLINDAMYCIN	Urinary System Disease	0.93
LUCINACTANT	Malignant Neoplasm of Other Additionally, Unspecified Major Salivary Glands	0.93
CINNARIZINE	Cancer	0.93
ETOPOSIDE	Artery Disease	0.93
L-ORNITHINE	Carcinoma	0.92
LUCINACTANT	Disorder Of Urinary System	0.92
IMATINIB	Heart Conduction Disease	0.91
L-ORNITHINE	Heart Disease	0.89
NELARABINE	Heart Conduction Disease	0.88
NIMODIPINE	Abscess Of Lung Additionally, Mediastinum	0.87
METHOTREXATE	Integumentary System Cancer	0.86
PREDNISOLONE	In Situ Neoplasms	0.85
MELATONIN	Cognitive Disorder	0.85
TEMOZOLOMIDE	Other Disorders of Urinary System	0.84
ANASTROZOLE	Malignant Neoplasm of Other Endocrine Glands Additionally, Related Structures	0.82
FLUOCINOLONE ACETONIDE	Other Diseases of Liver	0.79
AGALSIDASE β	Carbohydrate Metabolism Disease	0.77
CALCIUM ACETATE	Type 2 Diabetes Mellitus	0.75
CYSTEAMINE	Other Disorders of Carbohydrate Metabolism	0.74
VITAMIN C	Type 2 Diabetes Mellitus	0.74
L-CARNITINE	Autosomal Dominant Disease	0.70
IBUPROFEN	Cardiovascular System Disease	0.70

**Table 5:** Ten-fold cross validation accuracies for link prediction using RF, Gboost, and GNN in DDKG.

Methods	1	2	3	4	5	6	7	8	9	10	AUROC
RF	96.69	99.44	99.60	98.05	99.88	99.65	98.34	98.86	99.68	99.52	98.09
GB	92.10	97.12	99.80	91.60	99.69	96.83	97.07	94.86	97.32	98.39	96.19
GNN	95.55	99.36	95.56	95.42	98.62	99.22	97.98	95.18	99.86	100.00	97.70
GCN	99.75	100.00	99.75	99.872	99.87	100.00	99.75	100.00	100.00	99.87	99.19

**Table 6:** Graph theoretic measures for the GDKG and DDKG networks.

Network Measure	GDKG	DDKG
Spectral gap	37.5218	99.7221
Density	0.0221	0.0452
Average number of neighbors	26.423	13.345

## Data Availability

https://www.genelab.nasa.gov, accessed on 1 August 2021.

## Supplementary Materials

The following are available online at https://www.mdpi.com/article/10.3390/genes13030473/s1, Table S1: Network measures for the 473 genes in the Gene Disease Knowledge Graph (GDKG); Table S2: Network measures for the Drug nodes in the Drug Disease Knowledge Graph (DDKG).
