# Supplementary material for: Detection of Target Genes for Drug Repurposing to Treat Skeletal Muscle Atrophy in Mice Flown in Spaceflight
Source: Genes (Basel). 2022 Mar 8;13(3):473. doi: 10.3390/genes13030473 (PMC8953707; doi:10.3390/genes13030473)
Supplement: Supplementary file 1 [file genes-13-00473-s001.zip › genes-1577736-supplementary.pdf]

Table S1. Network measures for the 473 genes in the Gene Disease Knowledge Graph (GDKG).

Genes	Degree Distribution	Neighborhood Connectivity	Eigenvector Centrality	Subgraph Centrality
GNAS	171	64.95238095	0.061247409	5.20323×10^{30}
NEU1	168	65.33928571	0.069736994	6.74566×10^{30}
CSNK2B	166	69	0.067660944	6.35×10^{30}
BAG6	162	70.40666667	0.066949119	6.2171×10^{30}
FTO	152	71.10638298	0.06282446	5.47464×10^{30}
FXR2	149	69.73722628	0.059887443	4.97473×10^{30}
EIF4A1	149	69.73722628	0.059887443	4.97473×10^{30}
SUOX	1^{29}	73.32283465	0.05781322	4.63609×10^{30}
SMARCA4	1^{29}	73.16	0.057042437	4.5133×10^{30}
PRDX5	125	74.856	0.058613247	$4.765^{29} \times 10^{30}$
SPPL3	124	78.63709677	0.060269574	5.03842×10^{30}
EIF2S2	123	76.51219512	0.060038049	4.99978×10^{30}
ASH1L	123	88.9245283	0.05931309	4.87977×10^{30}
DDX6	121	62.95867769	0.046722122	5.20323×10^{30}
RPS26	117	48.30769231	0.035899678	1.78764×10^{30}
RAB5B	117	48.30769231	0.035899678	1.78764×10^{30}
PA2G4	114	79.59821429	0.055118759	4.21402×10^{30}
ABCF1	113	79.83168317	0.050157428	3.48954×10^{30}
RPL7A	108	81.50925926	0.054425	$6.3\ 5 \times 10^{30}$
SURF4	107	91.52631579	0.053615385	3.98728×10^{30}
MRPL4	103	52.45631068	0.034347373	1.63638×10^{30}
MTMR3	103	97.42857143	0.055131951	4.21604×10^{30}
MRPS18B	101	79.83168317	0.050157428	3.48954×10^{30}
FAF1	101	98.4494382	0.053760546	4.0089×10^{30}
PICK1	101	72.32222222	0.039143521	2.12529×10^{30}
ACTR2	100	100.0454545	0.054018587	4.04748×10^{30}
SMAD4	97	88.47252747	0.049546408	3.40504×10^{30}
PMPCA	93	106.5555556	0.053951696	4.03746×10^{30}
RPL18	93	83.58064516	0.04833176	2.36829×10^{30}
NRBP1	88	95.68604651	0.051190181	3.63472×10^{30}
RPS9	86	95.62857143	0.040744533	2.3027×10^{30}
LMAN2	85	105.4819277	0.053979513	4.04162×10^{30}
SAMM50	84	100.5	0.044542522	2.75199×10^{30}
PSMD13	84	61.66666667	0.03075417	1.31192×10^{30}
CUX1	83	103.9130435	0.044947966	2.80232×10^{30}
MAP2K1	82	110.6818182	0.045447749	2.86499×10^{30}
DNLZ	81	106.5555556	0.053951696	4.03746×10^{30}
RANGAP1	78	107.1052632	0.049938988	3.45921×10^{30}
CNEP1R1	78	87.28205128	0.041320756	2.36829×10^{30}
MTCH2	77	106.9733333	0.048081822	3.20671×10^{30}
ACO2	76	108.1369863	0.048953139	3.32398×10^{30}

POLR2E	76	92.67105263	0.042469675	2.36829×10^{30}
MAPKAPK5	73	105.2465753	0.046861906	2.57655×10^{30}
IGF2R	72	134.0784314	0.041501652	2.38907×10^{30}
COL4A2	71	112.1967213	0.040889503	2.31911×10^{30}
SPTBN1	70	104.4626866	0.043099323	2.57655×10^{30}
COL4A1	70	113.4029851	0.046439171	2.99135×10^{30}
CHMP1A	70	111.3571429	0.048322306	3.23887×10^{30}
TIMMDC1	70	53.31428571	0.022899714	7.27374×10^{29}
VEGFB	68	69.14705882	0.029773989	1.22962×10^{30}
ARPC2	67	105.4328358	0.043719679	2.65126×10^{30}
HCFC1	67	118.1875	0.046280893	2.97099×10^{30}
PPM1G	67	60	0.025356748	8.91836×10^{29}
GOPC	67	136.1636364	0.045568478	2.88023×10^{30}
TOM1	67	130.6491228	0.045680939	2.89446×10^{30}
TAX1BP1	66	114.4814815	0.037460858	1.9465×10^{30}
DMXL1	66	110.5238095	0.043166125	3.23887×10^{30}
ABCC9	65	117.1612903	0.044836925	2.78849×10^{30}
NDUFS3	65	67.12307692	0.02616828	9.49835×10^{29}
VDAC1	65	55.07692308	0.021406579	6.35613×10^{29}
MYH7	65	119.1129032	0.045625898	2.88749×10^{30}
NCBP1	65	129.6470588	0.04064183	2.2911×10^{30}
SLAIN2	63	114.5614035	0.039662096	2.18197×10^{30}
REST	63	116.65	0.042527265	2.50861×10^{30}
UQCC2	63	101.031746	0.038304953	2.06241×10^{30}
LARP4B	63	152.1428571	0.038863267	2.06241×10^{30}
SF3A3	62	130.1538462	0.040797436	2.30868×10^{30}
PKD2	61	136.6538462	0.043663338	2.64443×10^{30}
SND1	61	135.8305085	0.050096612	3.48109×10^{30}
IRF2BP1	60	100.3	0.036291701	1.82689×10^{30}
PTTG1	60	48.43333333	0.01720394	4.10538×10^{29}
MARK3	60	129.5172414	0.045132088	2.82533×10^{30}
RNF181	59	113.9152542	0.040704228	2.29814×10^{30}
PRMT5	58	140.6086957	0.038560157	2.06241×10^{30}
COG6	58	114.9285714	0.038754777	2.08328×10^{30}
DDA1	57	114.1578947	0.03960289	2.17546×10^{30}
DCAF12	57	158.8	0.043655999	2.64354×10^{30}
PDE4DIP	57	119.3508772	0.042179134	2.4677×10^{30}
KPNB1	56	122.3333333	0.039219072	2.1335×10^{30}
COPS6	56	71.16071429	0.024247173	8.15493×10^{29}
LLGL1	56	143.1136364	0.037574991	1.95837×10^{30}
MSL2	55	118.3018868	0.036588654	1.85691×10^{30}
ZKSCAN1	55	133.245283	0.042921168	2.55529×10^{30}
ZC3H18	55	156.3333333	0.042952315	2.559×10^{30}
EIF4B	54	140.952381	0.035524871	1.7505×10^{30}
TRIP11	53	129.6981132	0.042607277	2.51806×10^{30}

CTDSP1	52	126.4423077	0.040437973	2.26818×10^{30}
RANBP2	52	130.5961538	0.041950444	2.44102×10^{30}
ETF1	52	153.8	0.03665473	1.86362×10^{30}
EIF3H	52	142.3809524	0.036004937	1.79813×10^{30}
MRPL9	50	65.36	0.019456234	5.25068×10^{29}
GTF2I	49	80.59183673	0.024132093	6.35×10^{30}
SNRPC	48	140.2291667	0.040414075	2.2655×10^{30}
FEM1A	48	130.8333333	0.038172071	2.02111×10^{30}
NF2	48	138	0.040102593	2.23071×10^{30}
PSMD6	48	176.4736842	0.040747822	2.30307×10^{30}
USP24	47	148.5909091	0.039469325	2.16081×10^{30}
TRAK1	47	171.7714286	0.036298728	1.8276×10^{30}
RABEP1	47	140.4444444	0.037294732	2.2655×10^{30}
FKBP8	47	159.0810811	0.034965219	2.35786×10^{30}
RYK	47	156.1621622	0.034614165	2.35786×10^{30}
RPL8	45	165.6046512	0.043635755	2.64109×10^{30}
PRPF6	45	162.3023256	0.041992985	2.44597×10^{30}
DEF8	45	133.6666667	0.036373637	1.83515×10^{30}
ATG4D	45	147.3953488	0.037441563	1.94449×10^{30}
UBE3B	45	160.547619	0.04046323	2.27101×10^{30}
SP2	45	159.6046512	0.041229725	2.35786×10^{30}
CKAP5	45	158.1190476	0.040038435	2.35786×10^{30}
ZFP91	44	165.2195122	0.041521483	2.39135×10^{30}
NPR2	44	177.4285714	0.045962471	2.93025×10^{30}
FXR1	44	154.4285714	0.039173881	2.12859×10^{30}
CUL4A	44	144.547619	0.035273385	1.72581×10^{30}
ALKBH5	44	143.1136364	0.037574991	1.95837×10^{30}
CDC42	44	93.5	0.02391507	7.93307×10^{29}
SPNS1	43	161	0.039724379	2.18883×10^{30}
EIF5A	43	155.0540541	0.034066214	1.6097×10^{30}
PHACTR2	43	153.195122	0.037347297	1.93471×10^{30}
MRPS^{30}	43	173.0909091	0.033746552	1.57964×10^{30}
GSTM5	42	171.25	0.032571766	1.47157×10^{30}
ALS2	42	155.1666667	0.040068929	2.22697×10^{30}
TNNT3	42	132.5952381	0.033538853	1.56025×10^{30}
CUL1	42	173.05	0.042271442	2.47852×10^{30}
NUCKS1	42	88.95238095	0.022356415	6.9327×10^{29}
TRIP12	42	155.1666667	0.040068929	2.22697×10^{30}
QRICH1	42	151.875	0.035663515	1.76419×10^{30}
NFIX	42	171.725	0.041245411	2.35966×10^{30}
ASCC2	42	166.375	0.04034182	2.2574×10^{30}
HECTD1	42	155.1666667	0.040068929	2.22697×10^{30}
NAA10	42	68.16666667	0.017036415	4.02582×10^{29}
ANP32E	41	148.2307692	0.033977522	1.95837×10^{30}
SPAG7	40	88.325	0.021229175	6.25121×10^{29}

GTPBP1	40	201.75	0.033628316	1.56859×10^{30}
SLC25A11	40	88.325	0.021229175	6.25121×10^{29}
FAM53C	40	153.8	0.03665473	1.86362×10^{30}
GNS	40	201.75	0.033628316	1.56859×10^{30}
NONO	40	201.75	0.033628316	1.56859×10^{30}
ANAPC1	40	164.0526316	0.036423441	1.84018×10^{30}
PPP1R15B	40	201.75	0.033628316	1.56859×10^{30}
FOXO4	40	201.75	0.033628316	1.56859×10^{30}
GSPT1	40	148.2	0.035659453	1.76379×10^{30}
$SNAP^{29}$	39	195	0.033690059	1.57435×10^{30}
EIF4H	39	58.35897436	0.013410095	2.49437×10^{29}
SNX19	39	174.5405405	0.038961202	2.10554×10^{30}
NOC2L	39	203.4444444	0.032708443	1.48394×10^{30}
CRKL	39	195	0.033690059	1.57435×10^{30}
NDUFS2	39	65.92307692	0.014343859	2.85384×10^{29}
UFC1	39	65.92307692	0.014343859	2.85384×10^{29}
EIF3F	39	48.07692308	0.010655328	1.78764×10^{30}
PSMA3	38	190.1111111	0.041166331	2.35062×10^{30}
ACTR10	38	190.1111111	0.041166331	2.35062×10^{30}
TEX2	38	190.1111111	0.041166331	2.35062×10^{30}
USP4	38	165.0833333	0.034812927	1.68104×10^{30}
AP3S2	38	184.0833333	0.040151646	2.23617×10^{30}
OPA1	38	177.1142857	0.037645073	1.96569×10^{30}
RHOA	38	165.0833333	0.034812927	1.68104×10^{30}
UQCR11	38	158.4736842	0.036457749	2.35786×10^{30}
USP5	37	208.8518519	0.033711277	1.57633×10^{30}
TPI1	37	208.8518519	0.033711277	1.57633×10^{30}
PSMB6	37	72.56756757	0.015452455	3.31202×10^{29}
RPL4	37	160.7567568	0.035676782	1.76551×10^{30}
FIP1L1	37	173.2258065	0.031561853	1.38173×10^{30}
ELK4	37	168.5428571	0.034242274	1.62638×10^{30}
PARP1	37	208.7307692	0.032356507	1.45218×10^{30}
USP8	37	167.5142857	0.034804912	1.68027×10^{30}
FBXW2	37	68.32432432	0.014887025	65.92307692
MFN2	36	166.8823529	0.03320841	1.52966×10^{30}
RAF1	35	195.3125	0.037727655	1.97432×10^{30}
UBE2M	35	145.0857143	0.030410289	1.28274×10^{30}
MRPL10	35	99.6	0.021393908	6.3486×10^{29}
BRI3	35	165.2571429	0.034645834	1.66495×10^{30}
CHMP2A	35	145.0857143	0.030410289	1.28274×10^{30}
ADRM1	35	145.0857143	0.030410289	1.28274×10^{30}
ATG3	34	167.78125	0.031618316	1.38668×10^{30}
ZDHHC5	34	163.71875	0.030624029	1.30084×10^{30}
CRAT	34	179.34375	0.03411092	1.61393×10^{30}
DCTN2	34	95.02941176	0.019391184	5.21563×10^{29}

DHX36	34	206.0645161	0.038469584	2.05274×10^{30}
BAP1	34	168.8235294	0.034328001	1.63454×10^{30}
MAPK1	33	186.1290323	0.03370739	1.57597×10^{30}
YWHAE	33	189.3548387	0.034101319	1.61302×10^{30}
COPA	33	186.1290323	0.03370739	1.57597×10^{30}
HSP90AB1	33	184.9354839	0.034044412	1.60764×10^{30}
CTDSP2	33	186.1290323	0.03370739	1.57597×10^{30}
POLDIP2	33	56.81818182	0.010230319	1.57597×10^{30}
KLHL7	32	186.5333333	0.032784646	1.49087×10^{30}
ACTR1A	32	74.875	0.013452745	2.51027×10^{29}
CBX1	32	100.0625	0.019309103	5.17157×10^{29}
MPP6	31	178.4137931	0.030107788	1.25735×10^{30}
NCOR1	31	196.0967742	0.036874889	1.88608×10^{30}
TIMM44	31	210.137931	0.036836922	1.88219×10^{30}
AMFR	31	194.1034483	0.032747488	1.48749×10^{30}
CTBP1	31	210.137931	0.036836922	1.88219×10^{30}
AGGF1	31	210.137931	0.036836922	1.88219×10^{30}
CYC1	31	190.3793103	0.032135756	1.43244×10^{30}
KIF1C	31	210.137931	0.036836922	1.88219×10^{30}
LARP1B	31	210.137931	0.036836922	1.88219×10^{30}
ADSSL1	31	176.6129032	0.032611	1.47512×10^{30}
MEF2A	31	181.8064516	0.033836762	1.58809×10^{30}
AUH	31	197.6896552	0.034190547	1.62147×10^{30}
SGCB	31	210.137931	0.036836922	1.88219×10^{30}
RNF6	31	210.137931	0.036836922	1.88219×10^{30}
PACS2	31	181.8064516	0.033836762	1.58809×10^{30}
OXR1	31	181.8064516	0.033836762	1.58809×10^{30}
MED16	30	196.9285714	0.032666072	1.4801×10^{30}
DHX15	30	188.9285714	0.031124808	1.34373×10^{30}
AP3D1	30	198.6785714	0.032870564	1.49869×10^{30}
COX7A2L	30	61.56666667	0.010701196	1.58841×10^{29}
PPP6R3	30	219.3571429	0.037208892	1.9204×10^{30}
RPS18	30	157.5666667	0.028191441	1.10238×10^{30}
GDE1	29	203.7407407	0.031854172	1.40744×10^{30}
COQ9	29	206.037037	0.032854815	1.49726×10^{30}
KIFAP3	29	206.5925926	0.033121325	1.52164×10^{30}
NDUFA8	29	188.6551724	0.032610652	1.47508×10^{30}
LARS	29	203.7407407	0.031854172	1.40744×10^{30}
SNRPB	29	203.7407407	0.031854172	1.40744×10^{30}
ETFA	29	168.4827586	0.028580468	1.84018×10^{30}
JTB	28	201.75	0.033628316	1.56859×10^{30}
PGAM2	28	103.3214286	0.017519124	4.25719×10^{29}
TRAPPC1	28	201.75	0.033628316	1.56859×10^{30}
PITPNB	28	201.75	0.033628316	1.56859×10^{30}
MEF2D	27	214.52	0.031184881	1.34892×10^{30}

PSMB5	27	171.7777778	0.027098883	1.01859×10^{30}
RPL14	27	220.7037037	0.035965603	1.79421×10^{30}
CAPZB	27	79.66666667	0.012889074	2.30431×10^{29}
RAD23A	27	101.6666667	0.0166569	4.25719×10^{29}
ATP5B	26	227.4166667	0.031742113	1.39756×10^{30}
RPL3L	26	227.4166667	0.031742113	1.39756×10^{30}
DYM	26	227.4166667	0.031742113	1.39756×10^{30}
RPS11	26	109.4615385	0.017282587	4.143×10^{29}
KANSL1L	26	229.7083333	0.032841195	1.49601×10^{30}
HSPA9	26	212.5	0.029835653	1.23472×10^{30}
KLHDC2	26	223.125	0.031104012	1.34193×10^{30}
CCT2	26	227.4166667	0.031742113	1.39756×10^{30}
NDUFV1	26	229.7083333	0.032841195	1.49601×10^{30}
UBQLN4	26	223.125	0.031104012	1.34193×10^{30}
EFCAB14	26	229.7083333	0.032841195	1.49601×10^{30}
E2F6	26	121.4615385	0.018417602	4.70505×10^{29}
SEC13	26	212.5	0.029835653	1.23472×10^{30}
RPL13A	26	109.4615385	0.017282587	4.143×10^{29}
PSMC6	26	229.7083333	0.032841195	1.49601×10^{30}
SNF8	26	122.4615385	0.01925279	5.14145×10^{29}
COX6C	26	209.2692308	0.032471453	1.46252×10^{30}
RPS25	26	122.4615385	0.01925279	1.15072×10^{30}
ARF1	26	216.4583333	0.030574316	1.15072×10^{30}
CNOT4	26	216.0416667	0.030455479	1.27464×10^{30}
NR2C2	26	228.9166667	0.032084141	1.49601×10^{30}
EIF2AK1	25	238.2	0.035934788	1.79113×10^{30}
ABCF2	25	178.68	0.026059561	9.41959×10^{29}
IPO8	25	238.2	0.035934788	1.79113×10^{30}
ARFGEF1	25	238.2	0.035934788	1.79113×10^{30}
DHX8	25	238.2	0.035934788	1.79113×10^{30}
NGLY1	25	238.2	0.035934788	1.79113×10^{30}
UBR1	25	238.2	0.035934788	1.79113×10^{30}
PDS5A	25	238.2	0.035934788	1.79113×10^{30}
CC2D1A	25	238.2	0.035934788	1.79113×10^{30}
RLIM	25	238.2	0.035934788	1.79113×10^{30}
DENND5A	25	238.2	0.035934788	1.79113×10^{30}
USP7	25	238.2	0.035934788	1.79113×10^{30}
NRD1	25	215.3043478	0.028802884	1.15072×10^{30}
SMARCAL1	25	238.2	0.035934788	1.79113×10^{30}
IVNS1ABP	25	238.2	0.035934788	1.79113×10^{30}
DHX9	25	238.2	0.035934788	1.79113×10^{30}
SDHC	25	79.36	0.011093539	1.70702×10^{29}
SET	25	79.36	0.011093539	1.70702×10^{29}
CLCN4	25	238.2	0.035934788	1.79113×10^{30}
ZMYM5	25	238.2	0.035934788	1.79113×10^{30}

EEF1B2	25	217.24	0.032240968	1.44183×10^{30}
G3BP1	25	238.2	0.035934788	1.79113×10^{30}
EIF4ENIF1	25	238.2	0.035934788	1.79113×10^{30}
KDM7A	25	238.2173913	0.031989236	1.4194×10^{30}
NFU1	25	224.2608696	0.030314137	1.27464×10^{30}
PWP1	25	238.2	0.035934788	1.79113×10^{30}
SCN4A	25	238.2	0.035934788	1.79113×10^{30}
EIF4G1	25	238.2	0.035934788	1.79113×10^{30}
EIF1	25	212.52	0.031406076	1.36812×10^{30}
DENND4C	25	238.2	0.035934788	1.79113×10^{30}
LTN1	25	238.2	0.035934788	1.79113×10^{30}
CHMP4B	24	225.6666667	0.032276552	1.44501×10^{30}
NDUFB3	24	189.5833333	0.026575617	9.79636×10^{29}
PRCC	24	248.6818182	0.032252326	1.44285×10^{30}
MRPS31	24	137.375	0.018942288	4.97694×10^{29}
SLC2A4	24	248.6818182	0.032252326	1.44285×10^{30}
COX4I1	24	189.5833333	0.026575617	9.79636×10^{29}
EMC2	23	239.6190476	0.029455611	1.20347×10^{30}
RPL35	23	88.52173913	0.011201492	1.7404×10^{29}
ARPC5L	23	88.52173913	0.011201492	1.7404×10^{29}
MAP4K5	22	255.8	0.029937468	1.24316×10^{30}
ARL2	22	75.77272727	0.009530075	1.25977×10^{29}
RAB1B	22	90.40909091	0.011291552	1.7685×10^{29}
RNF14	22	260.45	0.030396719	1.2816×10^{30}
CHMP7	22	68.77272727	0.008238804	9.41513×10^{28}
MRPS21	22	77.68181818	0.009704774	1.25977×10^{29}
LSM4	22	100.7272727	0.012970636	4.25719×10^{29}
TMEM38A	21	263.6315789	0.029168445	1.18012×10^{30}
SGTA	21	263.6315789	0.029168445	1.18012×10^{30}
CAPNS1	21	263.6315789	0.029168445	1.18012×10^{30}
DNAJA3	21	263.6315789	0.029168445	1.18012×10^{30}
ADH5	21	92.0952381	0.010461128	1.51794×10^{29}
RNF185	21	100.4761905	0.011793849	1.92934×10^{29}
SKI	21	281.4210526	0.031138648	1.34492×10^{30}
SLTM	21	263.6315789	0.029168445	1.18012×10^{30}
FECH	21	270	0.030123963	1.2587×10^{30}
RPL37A	21	250.5238095	0.031069304	1.33894×10^{30}
FKBP9	21	247.6190476	0.030655684	1.33894×10^{30}
SLC35E1	21	263.6315789	0.029168445	1.33894×10^{30}
TMED10	20	110.9	0.013028903	2.35458×10^{29}
H1F0	20	71	0.007640864	8.09809×10^{28}
EDC4	20	249.0526316	0.027635893	1.05936×10^{30}
BYSL	19	128.1052632	0.014923381	3.0891×10^{29}
TMEM^{30}A	19	273.8421053	0.030508357	1.29103×10^{30}
PSME3	19	128.1052632	0.014923381	3.0891×10^{29}

UQCR10	19	128.1052632	0.014923381	3.0891×10^{29}
UBE2V1	19	128.1052632	0.014923381	3.0891×10^{29}
AURKAIP1	19	109.1052632	0.012277005	2.09066×10^{29}
RPL27A	19	128.1052632	0.014923381	3.0891×10^{29}
SDF4	19	109.1052632	0.012277005	2.09066×10^{29}
COA3	19	128.1052632	0.014923381	3.0891×10^{29}
VPS26A	19	128.1052632	0.014923381	3.0891×10^{29}
SUCLG1	19	271.9411765	0.026951254	1.00753×10^{30}
UBE2J2	19	109.1052632	0.012277005	2.09066×10^{29}
PSMA1	19	128.1052632	0.014923381	3.0891×10^{29}
NDUFB2	19	128.1052632	0.014923381	3.0891×10^{29}
GABARAPL2	17	111.3529412	0.010569496	1.54955×10^{29}
COLGALT1	17	102	0.009363501	1.21611×10^{29}
KAT2B	17	292.8125	0.02730014	1.03378×10^{30}
HIBADH	17	107.5882353	0.010752216	1.60359×10^{29}
TOLLIP	17	101.4705882	0.009906341	1.36121×10^{29}
NDUFA4	17	108.2941176	0.00994063	1.37065×10^{29}
VBP1	16	337.4	0.029484208	1.2058×10^{30}
DOLK	16	336.3333333	0.02939449	1.19848×10^{30}
NDE1	16	131.1875	0.011584312	1.86139×10^{29}
PECAM1	16	123.875	0.011017973	1.77×10^{29}
STUB1	15	304.0666667	0.026772848	9.9423×10^{29}
YWHAB	15	87.86666667	0.00704031	6.87514×10^{28}
CHCHD10	15	114.3333333	0.009355117	1.21394×10^{29}
SNRNP27	14	128.4285714	0.009828572	1.33992×10^{29}
SGPP1	14	132.3571429	0.010927653	1.65635×10^{29}
BCL2L2	14	128.4285714	0.009828572	1.33992×10^{29}
ADIPOR1	14	128.4285714	0.009828572	1.33992×10^{29}
RPS2	14	128.4285714	0.009828572	1.33992×10^{29}
NDUFA6	14	102.8571429	0.007922111	8.70522×10^{28}
RNPS1	14	128.4285714	0.009828572	1.33992×10^{29}
RNF103	13	99.38461538	0.006889343	6.58345×10^{28}
BROX	13	139.9230769	0.010075695	1.40815×10^{29}
OAZ1	13	128.8461538	0.009544127	1.33992×10^{29}
PSMC4	12	383.0833333	0.026809239	9.96935×10^{29}
FOXJ2	12	383.0833333	0.026809239	9.96935×10^{29}
EIF3D	12	383.0833333	0.026809239	9.96935×10^{29}
CSNK2A1	12	383.0833333	0.026809239	9.96935×10^{29}
OGDH	12	383.0833333	0.026809239	9.96935×10^{29}
PRDM4	12	383.0833333	0.026809239	9.96935×10^{29}
CCNI	12	383.0833333	0.026809239	9.96935×10^{29}
RTN2	12	383.0833333	0.026809239	9.96935×10^{29}
NDUFA10	12	383.0833333	0.026809239	9.96935×10^{29}
HRC	12	383.0833333	0.026809239	9.96935×10^{29}
ATP6V1E1	12	383.0833333	0.026809239	9.96935×10^{29}

ABCB7	12	383.0833333	0.026809239	9.96935×10^{29}
CAPN7	12	383.0833333	0.026809239	9.96935×10^{29}
UTP3	12	383.0833333	0.026809239	9.96935×10^{29}
MRPL44	12	383.0833333	0.026809239	9.96935×10^{29}
PPP2R5A	12	383.0833333	0.026809239	9.96935×10^{29}
SNW1	12	383.0833333	0.026809239	9.96935×10^{29}
MAP2K2	12	383.0833333	0.026809239	9.96935×10^{29}
FAM20B	12	383.0833333	0.026809239	9.96935×10^{29}
COPS4	12	383.0833333	0.026809239	9.96935×10^{29}
ZFR	12	383.0833333	0.026809239	9.96935×10^{29}
FBXO3	12	383.0833333	0.026809239	9.96935×10^{29}
ETNK1	12	383.0833333	0.026809239	9.96935×10^{29}
NDUFA9	12	383.0833333	0.026809239	9.96935×10^{29}
FITM1	12	383.0833333	0.026809239	9.96935×10^{29}
GALNT1	12	383.0833333	0.026809239	9.96935×10^{29}
UBXN4	12	383.0833333	0.026809239	9.96935×10^{29}
CDK16	12	383.0833333	0.026809239	9.96935×10^{29}
PIGT	12	383.0833333	0.026809239	9.96935×10^{29}
ACAD8	12	383.0833333	0.026809239	9.96935×10^{29}
VPS26B	12	383.0833333	0.026809239	9.96935×10^{29}
USP12	12	383.0833333	0.026809239	9.96935×10^{29}
TMBIM4	12	383.0833333	0.026809239	9.96935×10^{29}
NOMO1	12	383.0833333	0.026809239	9.96935×10^{29}
OGFR	12	383.0833333	0.026809239	9.96935×10^{29}
ABCE1	12	383.0833333	0.026809239	9.96935×10^{29}
VPS35	12	383.0833333	0.026809239	9.96935×10^{29}
SDHAF2	12	149.6666667	0.009901817	1.35997×10^{29}
DDB1	12	383.0833333	0.026809239	9.96935×10^{29}
UQCRFS1	12	383.0833333	0.026809239	9.96935×10^{29}
PRKACA	12	383.0833333	0.026809239	9.96935×10^{29}
SRP68	12	383.0833333	0.026809239	9.96935×10^{29}
HP1BP3	12	383.0833333	0.026809239	9.96935×10^{29}
FBXW5	12	383.0833333	0.026809239	9.96935×10^{29}
KHDRBS1	12	383.0833333	0.026809239	9.96935×10^{29}
DVL3	12	383.0833333	0.026809239	9.96935×10^{29}
TCP1	12	383.0833333	0.026809239	9.96935×10^{29}
NCL	12	383.0833333	0.026809239	9.96935×10^{29}
YME1L1	12	383.0833333	0.026809239	9.96935×10^{29}
PKM	12	383.0833333	0.026809239	9.96935×10^{29}
GLYR1	12	383.0833333	0.026809239	9.96935×10^{29}
HADHB	12	383.0833333	0.026809239	9.96935×10^{29}
RUFY1	12	383.0833333	0.026809239	9.96935×10^{29}
MDH2	12	383.0833333	0.026809239	9.96935×10^{29}
MXI1	12	383.0833333	0.026809239	9.96935×10^{29}
EEF1G	12	383.0833333	0.026809239	9.96935×10^{29}

MTA1	12	383.0833333	0.026809239	9.96935×10^{29}
FOXK2	12	383.0833333	0.026809239	9.96935×10^{29}
UBA1	12	383.0833333	0.026809239	9.96935×10^{29}
MIOS	12	383.0833333	0.026809239	9.96935×10^{29}
SSX2IP	12	383.0833333	0.026809239	9.96935×10^{29}
PHF10	12	383.0833333	0.026809239	9.96935×10^{29}
SERINC1	12	383.0833333	0.026809239	9.96935×10^{29}
CDH5	12	383.0833333	0.026809239	9.96935×10^{29}
VCP	12	383.0833333	0.026809239	9.96935×10^{29}
LONP1	12	383.0833333	0.026809239	9.96935×10^{29}
UBA3	12	383.0833333	0.026809239	9.96935×10^{29}
DCTN1	12	383.0833333	0.026809239	9.96935×10^{29}
GNPAT	12	383.0833333	0.026809239	9.96935×10^{29}
HDGF	12	150.9166667	0.010338784	1.48264×10^{29}
BCCIP	12	383.0833333	0.026809239	9.96935×10^{29}
BUB3	12	383.0833333	0.026809239	9.96935×10^{29}
ATP6AP1	12	383.0833333	0.026809239	9.96935×10^{29}
GHITM	12	383.0833333	0.026809239	9.96935×10^{29}
VDAC2	12	383.0833333	0.026809239	9.96935×10^{29}
PPA1	12	383.0833333	0.026809239	9.96935×10^{29}
KPNA6	12	383.0833333	0.026809239	9.96935×10^{29}
SRSF4	12	383.0833333	0.026809239	9.96935×10^{29}
RPL10A	12	150.9166667	0.010338784	1.48264×10^{29}
ALDH4A1	12	383.0833333	0.026809239	9.96935×10^{29}
UBQLN1	12	383.0833333	0.026809239	9.96935×10^{29}
TCOF1	12	383.0833333	0.026809239	9.96935×10^{29}
CENPB	12	383.0833333	0.026809239	9.96935×10^{29}
HADHA	12	383.0833333	0.026809239	9.96935×10^{29}
IDH3B	12	383.0833333	0.026809239	9.96935×10^{29}
NUPL1	12	383.0833333	0.026809239	9.96935×10^{29}
MTMR6	12	383.0833333	0.026809239	9.96935×10^{29}
RHBDD1	12	383.0833333	0.026809239	9.96935×10^{29}
CCT4	12	383.0833333	0.026809239	9.96935×10^{29}
ULK2	12	383.0833333	0.026809239	9.96935×10^{29}
PLAA	12	383.0833333	0.026809239	9.96935×10^{29}
KCMF1	12	383.0833333	0.026809239	9.96935×10^{29}
IMMT	12	383.0833333	0.026809239	9.96935×10^{29}
NDUFS1	12	383.0833333	0.026809239	9.96935×10^{29}
ECD	12	383.0833333	0.026809239	9.96935×10^{29}
HHATL	12	383.0833333	0.026809239	9.96935×10^{29}
PPP2R5D	12	383.0833333	0.026809239	9.96935×10^{29}
UHMK1	12	383.0833333	0.026809239	9.96935×10^{29}
ETFDH	12	383.0833333	0.026809239	9.96935×10^{29}
HDAC2	12	383.0833333	0.026809239	9.969351
CCDC90B	12	383.0833333	0.026809239	9.969351

DPF2	12	383.0833333	0.026809239	9.969351
TPCN1	12	383.0833333	0.026809239	9.969351
NDRG2	12	383.0833333	0.026809239	9.969351
DDX42	12	383.0833333	0.026809239	9.969351
DNAJC3	12	383.0833333	0.026809239	9.969351
STIM1	12	383.0833333	0.026809239	9.969351
RAB18	12	383.0833333	0.026809239	9.969351
HNRNPDL	12	383.0833333	0.026809239	9.969351
BAG1	11	128.9090909	0.007721052	8.26896×10^{28}
RPL19	10	154.9	0.008483177	9.98194×10^{28}
TERF2IP	10	169.1	0.009235879	1.18319×10^{29}
PEF1	10	154.9	0.008483177	9.98194×10^{28}
LSM12	10	124.8	0.006629217	9.98194×10^{28}
TUSC2	9	149.8888889	0.007254903	7.30064×10^{28}
TXNL4A	9	149.8888889	0.007254903	7.30064×10^{28}
PAM16	9	149.8888889	0.007254903	7.30064×10^{28}
MPC2	9	149.8888889	0.007254903	7.30064×10^{28}
TMBIM6	9	149.8888889	0.007254903	7.30064×10^{28}
TNNC1	8	152.75	0.00647825	5.82121×10^{28}
THAP11	8	75.875	0.003276712	1.48927×10^{28}
DNAJC^{30}	8	75.875	0.003276712	1.48927×10^{28}
PNO1	6	165.6666667	0.005483689	4.17103×10^{28}
NDUFB9	6	164.1666667	0.00543306	4.09437×10^{28}
ARL1	5	113	0.002970556	1.22398×10^{28}
RPS7	4	127	0.002646935	9.71817×10^{27}
PAIP2	4	127	0.002646935	9.71817×10^{27}
BUD31	1	4	1.07729E-05	1.60978×10^{23}

Table S2. Network measures for the Drug nodes in the Drug Disease Knowledge Graph (DDKG).

**Drug Names**	**Degree**	**Neighborhood Connectivity**	**Eigenvector Centrality**	**Subgraph Centrality**
DOXORUBICIN	21	10	3.01×10^{-15}	994,567.015
ARCITUMOMAB	76	10	1.24×10^{-14}	17,301,295.9
NELARABINE	21	10	3.03×10^{-15}	994,567.015
IFOSFAMIDE	21	10	3×10^{-15}	994,567.015
IXABEPILONE	21	10	3.01×10^{-15}	994,567.015
IMATINIB	21	10	2.96×10^{-15}	994,567.015
ETOPOSIDE	37	10	6.2×10^{-15}	4,284,550.57
NIMODIPINE	49	10	8.13×10^{-15}	7,458,879.19
TEMOZOLOMIDE	12	10	1.53×10^{-15}	251,605.014
METHOTREXATE	12	10	1.5×10^{-15}	251,605.014
CANAGLIFLOZIN	17	10	2.55×10^{-16}	25,038.3588
INSULIN REGULAR	17	10	2.62×10^{-16}	25,038.3588

INSULIN LISPRO	17	10	2.3×10^{-16}	25,038.3588
INSULIN ASPART	17	10	2.25×10^{-16}	25,038.3588
INSULIN GLARGINE	17	10	2.67×10^{-16}	25,038.3588
INSULIN, ISOPHANE	17	10	2.33×10^{-16}	25,038.3588
INSULIN GLULISINE	17	10	2.22×10^{-16}	25,038.3588
INSULIN DETEMIR	17	10	2.45×10^{-16}	25,038.3588
PRAMLINTIDE	17	10	2.36×10^{-16}	25,038.3588
L-CARNITINE	58	10	6.47×10^{-16}	272,705.747
TRANYLCYPROMINE	6	10	-3.73×10^{-18}	126.740606
FLUOXETINE	7	10	1.18×10^{-17}	151.497096
MAPROTILINE	7	10	-2.98×10^{-17}	151.497096
RISPERIDONE	6	10	-1.61×10^{-17}	126.740606
SERTRALINE	7	10	-2.5×10^{-17}	151.497096
PAROXETINE	7	10	8.68×10^{-18}	151.497096
LAMOTRIGINE	6	10	1.33×10^{-17}	126.740606
PHENELZINE	7	10	-5.5×10^{-18}	151.497096
VENLAFAXINE	6	10	-2.47×10^{-17}	126.740606
ISOCARBOXAZID	6	10	8.71×10^{-18}	126.740606
L-CARNITINE	58	10	2.26×10^{-15}	6,846,278.94
THIAMINE	35	10	2.31×10^{-15}	6,846,278.94
GALSULFASE	35	10	2.29×10^{-15}	6,846,278.94
IDURSULFASE	35	10	2.3×10^{-15}	6,846,278.94
ROSUVASTATIN	35	10	2.3×10^{-15}	6,846,278.94
CYSTEAMINE	35	10	2.31×10^{-15}	6,846,278.94
AGALSIDASE BETA	35	10	2.26×10^{-15}	6,846,278.94
VITAMIN C	35	10	2.32×10^{-15}	6,846,278.94
RIBOFLAVIN	35	10	2.3×10^{-15}	6,846,278.94
CALCIUM ACETATE	35	10	2.31×10^{-15}	6,846,278.94
KETOCONAZOLE	39	10	0.2236068	18,862,482.1
SULFISOXAZOLE	39	10	0.2236068	18,862,482.1
TINIDAZOLE	39	10	0.2236068	18,862,482.1
CHLOROQUINE	39	10	0.2236068	18,862,482.1
TETRACYCLINE	39	10	0.2236068	18,862,482.1
IVERMECTIN	39	10	0.2236068	18,862,482.1
PIPERAZINE	39	10	0.2236068	18,862,482.1
SURAMIN	39	10	0.2236068	18,862,482.1
DIETHYLCARBAMAZINE	39	10	0.2236068	18,862,482.1
ALBENDAZOLE	39	10	0.2236068	18,862,482.1
BUDESONIDE	22	10	2.46×10^{-16}	138,229.581
OLSALAZINE	22	10	2.48×10^{-16}	138,229.581
BALSALAZIDE	22	10	2.2×10^{-16}	138,229.581
HYDROCORTISONE	22	10	2.24×10^{-16}	138,229.581
SULFASALAZINE	22	10	2.25×10^{-16}	138,229.581

CERTOLIZUMAB PEGOL	22	10	2.27×10^{-16}	138,229.581
AZATHIOPRINE	22	10	2×10^{-16}	138,229.581
INFLIXIMAB	22	10	2.38×10^{-16}	138,229.581
GOLIMUMAB	22	10	2.22×10^{-16}	138,229.581
ADALIMUMAB	22	10	2.44×10^{-16}	138,229.581
L-ARGININE	50	10	8.85×10^{-15}	8,954,934.94
HYDRALAZINE	45	10	8.47×10^{-15}	8,223,630.15
MEMANTINE	9	9	1.51×10^{-15}	255,174.059
GALANTAMINE	1	5	7.07×10^{-17}	839.253743
PREDNISONE	54	10	9.27×10^{-15}	9,859,840.45
PREDNISOLONE	49	10	8.81×10^{-15}	8,890,290.51
WARFARIN	19	10	3.63×10^{-15}	1,523,624.8
DICHLORPHENAMIDE	7	10	1.24×10^{-15}	171,139.347
FLUOCINOLONE ACETONIDE	7	10	1.24×10^{-15}	171,139.347
LOTEPREDNOL	7	10	1.23×10^{-15}	171,139.347
L-ORNITHINE	43	10	8.26×10^{-15}	7,770,468.81
ANASTROZOLE	8	10	1.42×10^{-15}	226,794.168
CINNARIZINE	8	10	1.42×10^{-15}	226,794.168
VERAPAMIL	20	10	3.86×10^{-15}	1,681,914.24
N-ACETYL-D-GLUCOSAMINE	6	10	5.64×10^{-16}	37,755.8589
INDOMETHACIN	6	10	5.62×10^{-16}	37,755.8589
DIHYDROTACHYSTEROL	6	10	5.67×10^{-16}	37,755.8589
ERGOCALCIFEROL	6	10	5.52×10^{-16}	37,755.8589
IBUPROFEN	6	10	5.85×10^{-16}	37,755.8589
DESMOPRESSIN	6	10	5.85×10^{-16}	37,755.8589
ALENDRONATE	6	10	5.76×10^{-16}	37,755.8589
ENOXAPARIN	5	10	4.58×10^{-16}	25,126.9
HEPARIN	5	10	4.71×10^{-16}	25,126.9
UROKINASE	5	10	4.66×10^{-16}	25,126.9
STREPTOKINASE	5	10	4.81×10^{-16}	25,126.9
ACENOCOUMAROL	5	10	4.69×10^{-16}	25,126.9
ARGATROBAN	5	10	4.71×10^{-16}	25,126.9
NADROPARIN	5	10	4.65×10^{-16}	25,126.9
LUCINACTANT	16	10	3.16×10^{-15}	1,160,436.78
CLINDAMYCIN	16	10	3.15×10^{-15}	1,160,436.78
TELITHROMYCIN	16	10	3.16×10^{-15}	1,160,436.78
MELATONIN	1	10	-1.08×10^{-18}	2.95815542
AMPHETAMINE	1	10	1.04×10^{-17}	2.95815542
CITALOPRAM	1	10	1.01×10^{-17}	2.95815542
AMISULPRIDE	1	10	5.15×10^{-18}	2.95815542
FLUVOXAMINE	1	10	-9.29×10^{-18}	2.95815542
ISOPROPAMIDE	7	10	1.43×10^{-15}	230,272.897

DRONEDARONE	5	10	1.02×10^{-15}	116,165.404
